# Quantification of pathogen levels is necessary to compare responses to pathogen exposure: Comment on Davy et al. “The other white‐nose syndrome transcriptome”

**DOI:** 10.1002/ece3.4034

**Published:** 2018-05-08

**Authors:** Kenneth A. Field

**Affiliations:** ^1^ Department of Biology Bucknell University Lewisburg Pennsylvania

## Abstract

When studying host responses to the presence of pathogens, the pathogen levels should be verified within the samples. The RNA‐Seq samples from Davy et al. (2017) do not contain detectable *Pseudogymnoascus destructans* pathogen levels compared to other studies. Future studies will be necessary to determine how hosts resistant to white‐nose syndrome respond differently than susceptible hosts at the whole‐transcriptome level.

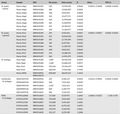

**Linked Article**: https://doi.org/10.1002/ece3.4035

White‐nose syndrome (WNS) is an epizootic disease that has killed millions of bats in North America (Blehert, 2012). WNS is caused by the psychrophile *Pseudogymnoascus destructans*, an ascomycete fungal pathogen (Gargas, Trest, Christensen, Volk, & Blehert, 2009; Lorch et al., 2011) that affects bats during hibernation. *P. destructans* can infect bats without causing mortality, as seen in Europe (Wibbelt et al., 2013; Zukal et al., 2016) and in some bats in North America (Frank et al., 2014; Lilley et al., 2016; Moore et al., 2018). An important question in the disease ecology of WNS is how hosts that are resistant or tolerant to infection respond differently than susceptible hosts. A recent paper in *Ecology and Evolution* (Davy et al., 2017) attempts to address this question by comparing the transcriptomic responses of the WNS‐resistant *Myotis myotis* to the WNS‐susceptible *M. lucifugus*. This study demonstrated that *M. myotis* are resistant to infection under the same conditions that *M. lucifugus* are susceptible to infection and under which they develop WNS. Davy et al. further reported that there was no differential expression of genes associated with immune responses in exposed *M. myotis* bats, which, they claimed, indicated that immune responses do not drive tolerance of *P. destructans*. However, it needs to be clarified that this study was not able to compare gene expression responses of these two species to *P. destructans* exposure because the *M. myotis* samples analyzed were no longer infected with the pathogen.

Although the *M. myotis* were exposed to *P. destructans* in this study, they were not apparently infected at the time that the tissue samples were collected. This is clearly indicated in the results that describe that the *M. myotis* bats did not exhibit any signs of WNS and that only three of the eight swabs contained detectable *P. destructans* DNA. However, at least one of these swabs had a *C*_t_ value (40.068) that is typically below the detection limit of this assay (Muller et al., 2012). Without the use of a standard to quantify the number of *P. destructans* conidia that this *C*_t_ value represents, it is not possible to judge whether any of these bats were positive. It was also noted in this paper that the gene expression patterns determined by RNA‐Seq were not correlated with whether the bat had a PCR “positive” swab.

This observation led me to investigate whether the *M. myotis* samples from *P. destructans*‐exposed bats contained fungal pathogen RNA in the samples themselves. Because *P. destructans* is a eukaryotic pathogen, it is possible to use the Poly(A)‐selected RNA‐Seq data to measure pathogen level in each sample. Using the data from this study (Davy et al., 2017) in the Sequence Read Archive, I compared the levels of *P. destructans* transcripts to other published (Field et al., 2015) and unpublished datasets (Table 1). For this analysis, the RNA‐Seq data were quality trimmed and then the reads were mapped to the combined transcriptomes of *M. lucifugus* and *P. destructans* using Kallisto (Bray, Pimentel, Melsted, & Pachter, 2016). The read counts without normalization were then totaled separately for all *M. lucifugus* and *P. destructans* transcripts. The results shown in Table 1 demonstrate that there is no difference in the numbers of *P. destructans* reads in either the unexposed or the exposed *M. myotis* groups from the Davy et al. study. The “Mymy‐Pos” samples had 314 ± 89 *P. destructans* counts, and the “Mymy‐Neg” samples contained 390 ± 87 *P. destructans* counts. In both groups, this represents about 0.003% of the reads that mapped to *M. lucifugus* transcripts in each sample. This can be compared to the pooled *M. lucifugus* data (from the supplemental information of Davy et al.) that contained 1.6% and 3.8% of the reads that mapped to *P. destructans* relative to *M. lucifugus*. The results from the *M. lucifugus* samples are similar to what we found in our own study of wild‐infected *M. lucifugus* (Reeder et al., 2017) and a single WNS‐affected *M. myotis* sample that is present in the Sequence Read Archive (Table 1). From these results, I conclude that the *M. myotis* tissue samples used for the Davy et al. RNA‐Seq study did not contain *P. destructans*.

**Table 1:** Comparison of read counts for host and pathogen in tissue samples from bats

Group	Sample	SRA	Pd counts	Mylu counts	%	Mean	95% CI
*M. myotis* control	Mymy‐Neg1	SRR5676387	549	12,035,052	0.0046	0.0034 ± 0.0008	0.0028–0.0040
	Mymy‐Neg2	SRR5676386	286	12,062,807	0.0024		
	Mymy‐Neg3	SRR5676400	338	9,978,393	0.0034		
	Mymy‐Neg4	SRR5676390	322	12,677,171	0.0025		
	Mymy‐Neg5	SRR5676392	460	11,990,000	0.0038		
	Mymy‐Neg6	SRR5676398	423	11,969,992	0.0035		
	Mymy‐Neg7	SRR5676399	419	10,769,437	0.0039		
	Mymy‐Neg8	SRR5676391	320	11,362,077	0.0028		
*M. myotis* exposed	Mymy‐Pos1	SRR5676394	259	9,555,456	0.0027	0.0029 ± 0.0007	0.0024–0.0034
	Mymy‐Pos2	SRR5676393	213	10,063,022	0.0021		
	Mymy‐Pos3	SRR5676389	391	11,754,594	0.0033		
	Mymy‐Pos4	SRR5676388	185	8,237,926	0.0022		
	Mymy‐Pos5	SRR5676384	396	11,387,437	0.0035		
	Mymy‐Pos6	SRR5676401	408	9,832,452	0.0042		
	Mymy‐Pos7	SRR5676385	376	12,688,167	0.0030		
	Mymy‐Pos8	SRR5676397	284	11,991,073	0.0024			*M. lucifugus*
Mylu‐Neg1	SRR5676383	1,807	30,410,530	0.0059				
Mylu‐Neg2	SRR5676382	1,628	22,868,241	0.0071				
Mylu‐Pos1	SRR5676396	535,636	33,544,637	1.5968				
Mylu‐Pos2	SRR5676395	680,954	17,923,428	3.7992		
Mymy‐WNS	SRR4448951 SRR4448179	830,869	58,045,617	1.4314
Uninfected *M. lucifugus*	SSD011MYUN	SRR1869462	453	8,506,157	0.0053	0.0116 ± 0.0096	0.0032–0.0200
	SSD064MYUN	SRR1916834	368	7,515,264	0.0049		
	SSD075MYUN	SRR1916836	2,047	7,414,215	0.0276		
	SSD090MYUN	SRR1916839	840	6,349,779	0.0132		
	SSD114MYUN	SRR1916841	482	7,146,433	0.0067		
WNS *M. lucifugus*	KYMYLU06W	SRR1916825	157,269	9,337,975	1.6842	1.8568 ± 0.4233	1.518–2.195
	KYMYLU07W	SRR1916826	199,228	9,413,460	2.1164		
	KYMYLU11W	SRR1916827	155,828	9,341,624	1.6681		
	KYMYLU19W	SRR1916842	196,732	8,172,142	2.4073		
	KYMYLU23W	SRR1916830	133,935	6,513,932	2.0561		
	KYMYLU39W	SRR1916832	101,217	8,374,589	1.2086		

It is possible that other areas of the bat wing were infected with the pathogen but not the particular tissue used for the RNA‐Seq study, although this paper indicates that the whole wing was used for RNA extraction. Also, the very low to negative PCR results indicate that it is more likely that these individuals were simply not infected with *P. destructans*. In an unpublished study, I have examined whether gene expression patterns vary between adjacent tissues that are uninfected or infected with *P. destructans*. UV fluorescence (Turner et al., 2014) was used to identify *P. destructans*‐positive and *P. destructans*‐negative sites in *M. lucifugus* wing tissue from bats infected with *P. destructans* in captivity. The 12 UV‐negative biopsies had low levels of *P. destructans* reads in the RNA‐Seq data (0.13% ± 0.15% of mapped reads) while 10 of the 12 the UV‐positive biopsies had higher levels of *P. destructans* reads (4.17% ± 3.27% of mapped reads). When I compared host gene expression of the *P. destructans*‐negative to the *P. destructans*‐positive samples after the bats aroused from torpor, I found that they were dramatically different, indicating that uninfected tissue adjacent to areas of infection does not show the same patterns of gene expression as the areas of infection. In order to measure how gene expression is affected by *P. destructans* exposure, the RNA‐Seq samples must have detectable infection levels.

The Davy et al. study acknowledges this limitation when it states, “*M. myotis* experienced extremely limited fungal growth and did not exhibit symptoms of WNS.” This would not be a major concern if the paper simply reported the *M. myotis* transcriptome without any reference to WNS. However, the title of the paper indicates that it is studying “the other white‐nose syndrome transcriptome.” How is it possible to study a WNS transcriptome without WNS? The title also states that “Tolerant and susceptible hosts respond differently to the pathogen *Pseudogymnoascus destructans*” but the data clearly show that the “tolerant” hosts were not actually exposed to and thus responding to the pathogen. The following statement from the discussion clearly implies that the authors expected a response to the pathogen even though there was no pathogen present: ”Gene expression by tolerant *M. myotis* in response to *P. destructans* differs from that described in susceptible, North American *M. lucifugus* (Field et al., 2015; Supporting information). We detected no immune response to infection in tolerant *M. myotis*; in fact, we detected no substantial response to the pathogen at all.” The *M. myotis* bats had already cleared the *P. destructans* infection, presumably several weeks earlier during hibernation, if the infection was ever established. The obvious explanation for the lack of a response to *P. destructans* in the *M. myotis* samples is that there was no pathogen present in these samples. The resistance of *M. myotis* to *P. destructans* infection that underlies the Davy et al. study is a very interesting observation that should not be overlooked. This may be similar to what we have observed in *Eptesicus fuscus* (Moore et al., 2018), North American bats that are resistant to WNS (Frank et al., 2014).

Future studies should take care to quantify levels of infection in the RNA‐Seq samples directly to verify that samples from bats exposed to *P. destructans* are actually infected. Then, we may finally learn whether the secret to surviving WNS lies in host transcriptomic responses.
